# Thiophene Derivatives as Ligands for Highly Luminescent and Stable Manganese-Doped CsPbCl_3_ Nanocrystals

**DOI:** 10.3389/fchem.2022.849801

**Published:** 2022-03-01

**Authors:** Qian Wang, Long Gao, Chenxi Yu, Meng Wang, Lijie Gou, Jiaqi Zhang

**Affiliations:** Key Laboratory of Automobile Materials, Ministry of Education, College of Materials Science and Engineering, Jilin University, Changchun, China

**Keywords:** Mn-doped CsPbCl_3_, ligand, thiophene derivative, perovskite, nanocrystal

## Abstract

Ligands on the surface of perovskite nanocrystals are important to stabilize the nanocrystal structure. However, the research of ligands on Mn^2+^ ion-doped CsPbCl_3_ nanocrystals (Mn: CsPbCl_3_ NCs), a promising candidate family for the lightning community, is relatively rare. Here, we demonstrate a new ligand modification strategy for preparing high-quality Mn: CsPbCl_3_ NCs by a simple hot-injection method. Thiophene derivative, for the first time, is applied as ligands for perovskite nanocrystals. The new ligands of thiophene derivatives passivate defects on the surface of NCs and enhance optical properties, originating from the sulfur in thiophene additives binding to the uncoordinated lead ions. The photoluminescence quantum yield of the modified Mn: CsPbCl_3_ NCs is 93% in comparison with 46% of the pristine counterparts, whose value is the highest to date for ligand-modified Mn: CsPbCl_3_ NCs. Meanwhile, the thermal, storage, and purification stability are also significantly improved. The performance of related LEDs is also investigated.

## 1 Introduction

In recent years, all-inorganic lead halide perovskite nanocrystals (NCs) CsPbX_3_ (X = Cl, Br, I) have attracted great attention due to their high photoluminescence efficiency, tunable bandgap, high color purity, strong light absorption, and high carrier mobility (Yang et al., 2019a, 2020; Cao et al., 2020; Sun et al., 2020). In addition, compared with the organic–inorganic hybrid methylamine lead halide perovskite, they have higher light, thermal, and humidity stability, which all have spurred their applications in solar cells, photodetectors, light-emitting diodes, *etc* (Liu Y. et al., 2018; Lu et al., 2018). Among all inorganic lead perovskite materials, the photoluminescence quantum yields (PLQYs) of the green CsPbBr_3_ and red CsPbI_3_ NCs have reached above 90% (Zhang et al., 2018; Zhang et al., 2021 Y.), but the PLQY of blue–violet emission CsPbCl_3_ NCs is relatively low. The wide bandgap of CsPbCl_3_ nanocrystals (3.0 eV) can facilitate the formation of deep-level defects such as chlorine vacancies (V_Cl_) and surface segregation of Pb, affecting the lattice structure and decreasing the luminescence performance of CsPbCl_3_ nanocrystals, which greatly limit further application of all-inorganic CsPbCl_3_ materials (Huang et al., 2016; Wei et al., 2019; Román-Vázquez et al., 2020).

One main direction of current research on CsPbCl_3_ is the introduction of Mn ions into CsPbCl_3_ (Parobek et al., 2016; Das Adhikari et al., 2017; Liu et al., 2017). The doping of Mn^2+^ provides efficient dual-emission from Mn^2+^ ions (around 600 nm) and the host (around 400 nm), and NCs show an orange–red light which originates from the energy transfer from host perovskite to Mn^2+^ and contributes to the *d–d* transition between the ^4^T_1_–^6^A_1_ configurations. Mn dopants reduce the hazard of Pb and also introduce new optical properties, which have potential in further applications. However, the PLQY of Mn-doped perovskite NCs is still low, and the poor stability also needs to be resolved.

Currently, ion doping and ligand modification are two main strategies to improve the PLQY and the stability of perovskite NCs (Luo et al., 2019; Gao et al., 2021). Specifically for Mn: CsPbCl_3_ NCs, in terms of ion doping, alkaline earth metals (Ca^2+^, Mg^2+^, and Ba^2+^) (Liu W. et al., 2018; Song et al., 2020), transition metals (Ni^2+^, Cd^2+^, and Cu^2+^) (Wang et al., 2019a; Rana et al., 2019; Xing et al., 2019; Zhao Y. et al., 2020; Cao et al., 2020; Ricciarelli et al., 2020; Zheng et al., 2020; Zhou et al., 2020; Zhang R. et al., 2021), and rare-earth ions (Yb^3+^, Eu^2+^, and Tm^3+^) (Wang et al., 2019b; Milstein et al., 2019; Zhao J. et al., 2020) have been introduced into perovskite NCs to effectively passivate defects and improve PLQYs. Recently, it was revealed that doping Cu^2+^ (Zheng et al., 2020) or Ni^2+^ (Xing et al., 2019) into Mn^2+^: CsPbCl_3_ NCs could greatly enhance the PLQY to 70%, and treating Cd^2+^ ions can enhance the PLQY from 15% to 85% (Zhao Y. et al., 2020). For ligand modification, suitable surface ligands are essential for perovskites, which not only enhance the PL emission but also improve the stability. However, most of the reports for Mn-doped CsPbCl_3_ focus on ion doping, but few focus on the ligand modification. Sun et al. (2020) introduced dodecyl dimethylammonium chloride (DDAC) as a ligand into Mn: CsPbCl_3_, and proved that DDAC partially replaced OA and OAm ligands, while PLQY reached 90% and achieved a good performance. Luo et al. (2021) used sulfonate ligands to tune the dual-color emission of Mn: CsPbCl_3_ NCs. Nevertheless, the potential of more effective ligands, such as thiophene derivatives, remains rather unexplored. In this respect, recent literatures on high-performance perovskite solar cells (PSCs) using thiophene additives (Wen et al., 2020; Chen et al., 2021; Choi et al., 2021; Guo et al., 2021; Ren et al., 2021) to passivate the defects of perovskite films are highly encouraging. Consequently, we envision that thiophene additives have the potential to be further applied as ligands in the synthesis process of all-inorganic perovskite NCs.

Herein, we propose a facile strategy to synthesize highly efficient Mn: CsPbCl_3_ NCs by introducing thiophene derivatives 3-thienylboronic acid (TBA) as ligands in the hot-injection synthesis process, which extremely boosts the performance of perovskite NCs. The TBA passivates defects on the surface of the perovskites and enhances optical properties, originating from the sulfur in thiophene additives binding to the uncoordinated lead ions. The PLQY is enhanced from 46% in Mn: CsPbCl_3_ to 93% in TBA-Mn: CsPbCl_3_, whose value, to the best of our knowledge, is the highest one reported to date for ligand-modified Mn: CsPbCl_3_ NCs (Das Adhikari et al., 2017; Sun et al., 2020; Luo et al., 2021). Meanwhile, the thermal, storage, and purification stability are also significantly improved. The LED devices were also fabricated by employing the TBA-Mn: CsPbCl_3_ NCs as emitting materials, which obtain a high luminescence of 11160 cd m^−2^ at 3.7 V.

## 2 Materials and Methods

### 2.1 Materials

Cesium carbonate (CsCO_3_, 99.995%), oleic acid (OA, 90%), and 1-octadecene (ODE, 90%) were purchased from Sigma-Aldrich. Lead chloride (PbCl_2_, 99.999%), manganese chloride (MnCl_2_, 99%), oleylamine (OAm, 70%), and 3-thienylboronic acid (TBA, 98%) were purchased from Aladdin. Methyl acetate was obtained from Macklin, and toluene and n-hexane were provided by Tianjin Fuyu Chemical Co., Ltd.

### 2.2 Synthesis of Mn: CsPbCl_3_ and TBA:Mn:CsPbCl_3_

For the preparation of Cs–oleate, 814 mg CsCO_3_, 2.5 ml OA, and 30 ml ODE were added into a 100-ml three-necked flask. After vacuuming and nitrogen filling repeatedly, the mixture was heated to 120°C and kept dry for 1 h under vacuum, then heated to 150°C and flushed with nitrogen until a clear solution was obtained, and finally stored in a refrigerator.

For the synthesis of perovskite, in our experiment, the molar feed ratio of Pb–Mn was fixed to 1:1. 52.3 mg (0.188 mol) of PbCl_2_, 23.6 mg (0.188 mol) of MnCl_2_, and 10 ml of ODE were added into a 50 ml three-necked flask. After repeated vacuuming and nitrogen filling, the mixture was heated to 120°C. After keeping it dry at 120°C for 1 h under vacuum, 1 ml OAm and 1 ml OA were injected into the flask at the same temperature. When the solution turned clear, the temperature was increased to 180°C and 1 ml Cs–oleate was injected rapidly. After 5 seconds, the reaction mixture was cooled to room temperature in an ice water bath. For TBA:Mn:CsPbCl_3_, 52.3 mg (0.188 mol) of PbCl_2_, 23.6 mg (0.188 mol) of MnCl_2_, 48 mg TBA (0.376 mol), and 10 ml of ODE were added into a 50 ml three-necked flask. The remaining experimental steps were the same as in the Mn: CsPbCl_3_ NCs synthesis. The reaction mixture was centrifuged for 10 min at 5000 rpm. The supernatant was discarded and the precipitate was redispersed in 2 ml of hexane, and then stored in the refrigerator. For further purification, methyl acetate was added to the solution with a volume ratio of 1:2 and the precipitate was centrifuged for 10 min at 10000 rpm. The nanocrystals were washed three more times with methyl acetate and hexane. The final products were dispersed in hexane for further measurements.

### 2.3 Fabrication of LED Devices

For WLED device fabrication, UV (365 nm) LED chips were used to excite the orange Mn: CsPbCl_3_ (600 nm). The precipitate was dispersed in the toluene solvent after centrifugation and shaken well. The mixture was added into PMMA/toluene solvent. After that, 0.3 ml of the mixed solution was added dropwise to a UV-LED. The chips were placed on a hot plate (60°C) to evaporate the toluene, and the LEDs were fabricated.

### 2.4 Characterization

XRD patterns were collected with a Bruker D8 Advance diffractometer with Cu Kα1 radiation (λ = 1.54178 Å). The PL spectra and PL decay curves of the NCs were recorded on an FLS980-STM Edinburgh fluorescence spectrometer. The PLQY was directly tested with a spectrophotometer (FLS980) equipped with an integrating sphere. UV-visible absorption spectra were measured with a PerkinElmer Lambda 3600 UV-vis-NIR spectrometer. The TEM and HRTEM images were collected with an electron microscope (JEOL, JEM-2100F) at 200 kV. Energy-dispersive X-ray spectroscopy (EDX) measurements were recorded in an SEM instrument (SU- 8010) with an Oxford X-Man50 part to obtain the NC elemental mapping. FTIR spectra were tested with a NICOLET 6700 FTIR spectrometer. ^1^H-NMR investigation was conducted for the dispersion of NC powders in CDCl_3_ on Bruker 400 MHz NMR spectrometer. XPS spectra were recorded by a Kratos Axis Super DLD spectrometer. The CPCM-TBA and CPCM NCs dispersed in hexane solution were heated on a hot plate at 80°C under 60% humidity to observe their thermal stability, which was recorded by using an Ocean Optics spectrometer. The NCs were dropped on silicon wafers and stored in a dark drawer at room temperature to evaluate the storage/shelf stability. The EL spectra of the WLEDs were recorded by a PR650 SpectraScan spectrophotometer (Photo Research) in air and at RT.

## 3 Results and Discussion

**FIGURE 1:**
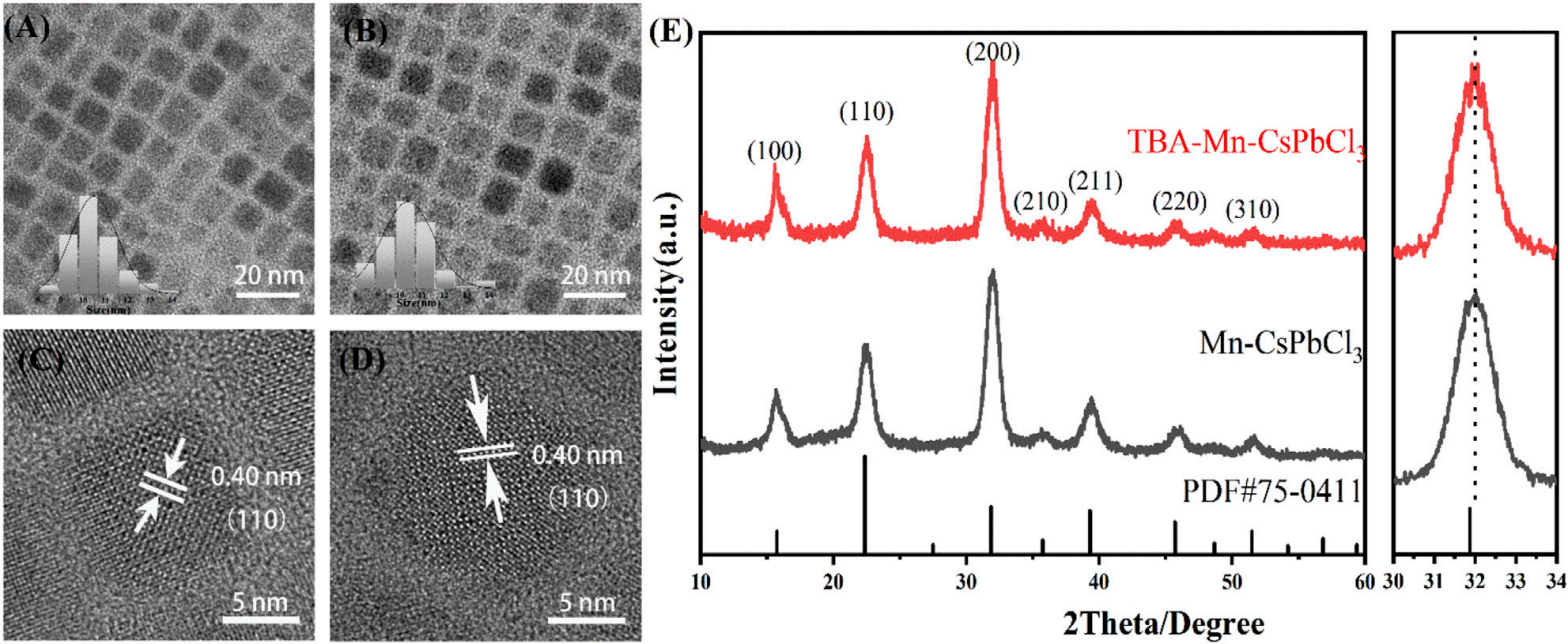
**(A)** TEM and **(C)** HRTEM images of CPCM NCs. **(B)** TEM and **(D)** HRTEM images of CPCM-TBA NCs. The insets of **(A)** and **(C)** are the histograms of particle size distribution. **(E)** XRD patterns of CPCM and CPCM-TBA.

High-quality Mn-doped CsPbCl_3_ NCs with 3-thienylboronic acid (TBA) were synthesized by the hot-injection method (Yang et al., 2020) (details are shown in Supplementary Figure S1). TBA was applied as the additional thiophene derivative ligand during the nanocrystal formation process. First, we explored the impact of TBA introduction on the morphology and structure of the perovskite NCs. Figures 1A,B show transmission electron microscopy (TEM) images of the as-prepared Mn-doped CsPbCl_3_ (CPCM) and TBA-modified Mn: CsPbCl_3_ (CPCM-TBA), which both exhibit cubic shape, with crystal sizes of 10.37 ± 1.41 nm and 10.48 ± 1.01 nm, respectively. Corresponding high-resolution TEM (HRTEM) images of CPCM and CPCM-TBA NCs both show high crystallinity and the same interplanar distance of 0.40 nm, which matched the (110) lattice plane (Figures 1C,D), elucidating that TBA did not cause lattice structure change of NCs. X-ray diffraction (XRD) patterns (Figure 1E) show main diffraction peaks at 15.7°, 22.5°, 32°, 39.4°, and 45.7° for (100), (110), (200), (211), and (220), respectively, which confirms that both the NCs exhibit a cubic perovskite structure. For the CPCM, a slight peak shift to a higher diffraction angle was observed in reference to CsPbCl_3_ (PDF# 75-0411), which is ascribed to the partial replacement of Pb^2+^ (∼1.33 Å) by Mn^2+^ (∼0.97 Å) (Liu et al., 2019). Compared with CPCM, the TBA-involved samples did not cause peak shift, indicating that both samples maintain the same nanocrystalline structure, and TBA may not change the stoichiometry of Mn and Pb. Consequently, the aforementioned results prove that the TBA as ligands has negligible influence on the lattice structure and the morphology of CPCM NCs.

**FIGURE 2:**
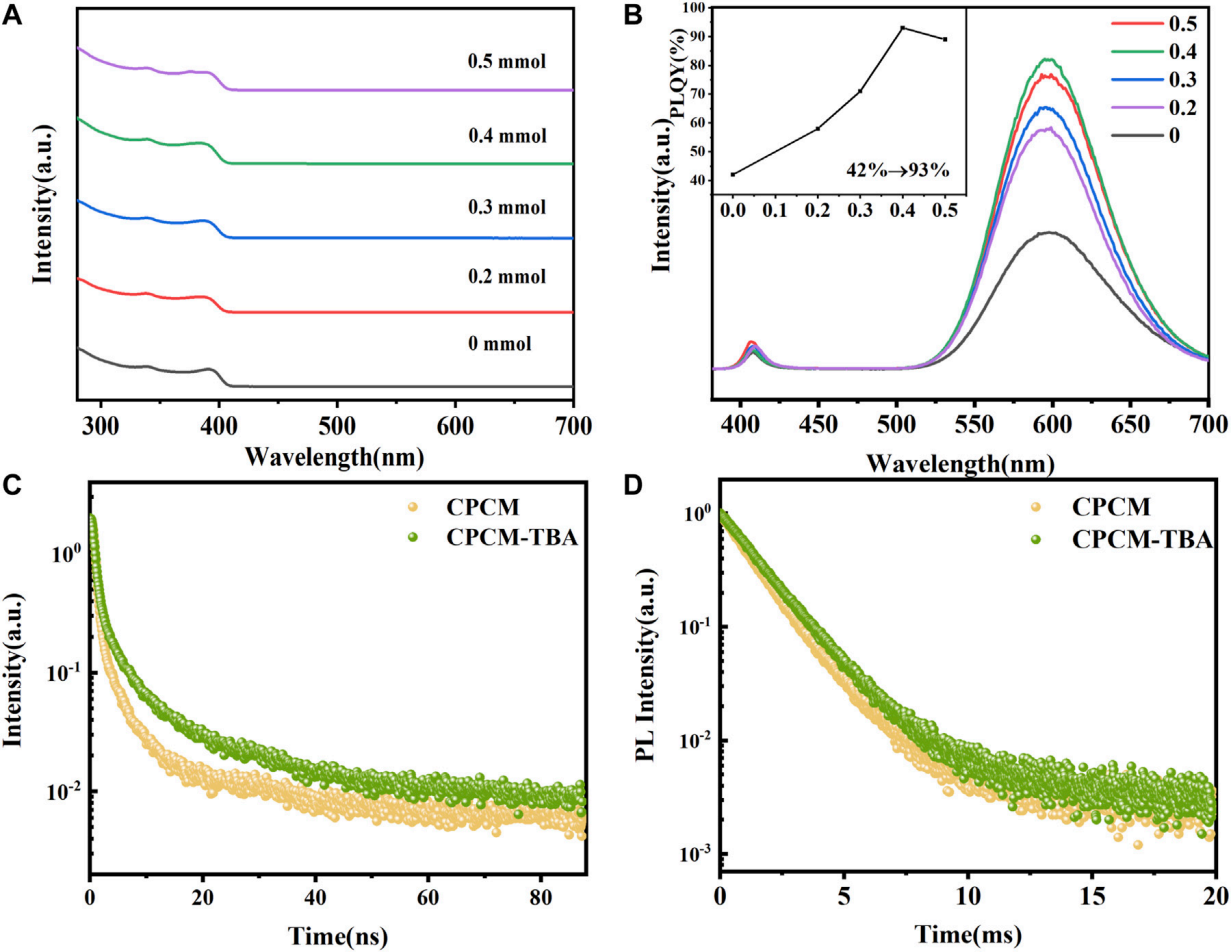
Absorption **(A)** and emission spectra **(B)** of CPCM NCs with 0–0.5 mmol of TBA; time-resolved PL decay of **(C)** exciton and **(D)** Mn^2+^ emission of the CPCM-TBA NCs.

We further investigated the influence of TBA addition on optical properties. The UV-visible absorption spectra of CPCM with different amounts of TBA are shown in Figure 2A. The exciton absorption peaks of all samples appear at 390 nm. The band-edge absorption position did not shift in an obvious manner with the addition of TBA, indicating the negligible influence of TBA on the electronic structure of CPCM. We also did not detect new absorption bands of Mn^2+^ for all the samples because the spin-forbidden *d–d* transition of Mn^2+^ is much weaker than the band-edge absorption (Parobek et al., 2016). The photoluminescence emission spectra were collected under 365 nm excitation, as shown in Figure 2B. Dual-color emission was observed for all samples. The PL spectrum of CPCM shows a narrow peak at 408 nm and a broad peak at 597 nm, which are attributed to the intrinsic exciton radiative recombination of CsPbCl_3_ NCs and Mn^2+^ (^4^T_1_→^6^A_1_) emission, respectively (Liu et al., 2017; Wang Shipping et al., 2020b). The exciton emission only experiences a little enhancement with the addition of TBA. However, the PL intensity of Mn^2+^ emission significantly increases with TBA content from 0 to 0.4 mmol, which may be attributed to the defect passivation of NCs by TBA addition. The passivation may come from the S atoms in TBA forming Pb–S bonds to decrease the chloride vacancies and lead dangling bond (He et al., 2021). The peak positions of both exciton and Mn^2+^ emissions did not observe an obvious shift, further reflecting that the Mn:Pb actual ratio may not change with the TBA addition, which was proved by the aforementioned XPS and EDX results (Supplementary Tables S1, S2 and Supplementary Figure S2). The inset of Figure 2B shows the PLQYs of NCs by adding TBA, whose trend is the same as the PL intensity of Mn^2+^. The PLQY remarkably improved from 43% (0 mmol) to a maximum of 93% (0.4 mmol) with the addition of TBA, whose value is the highest one reported to date for Mn: CsPbCl_3_ NCs. However, the intensity of Mn^2+^ emission and the PLQY start to reduce over 0.4 mmol TBA concentration, which may originate from the excess substitution of TBA ligands and may bring an unexpected change of the NC structure (Sun et al., 2020; Shao et al., 2020).

The PL decay curves of excitons and Mn^2+^ ions are shown in Figures 2C,D. The PL decay profiles of excitonic emission of the NCs can be fitted with two-exponential decay, and Mn^2+^ prefers single-exponential decay (Li et al., 2019). The average PL lifetimes were estimated using Eq. 1 (Li et al., 2019),

$$\tau_{ave}=\frac{\sum A_{i}\tau_{i}^{2}}{\sum A_{i}\tau_{i}},$$ (1)

where A_i_ and τ_i_ are the weights and time components of the exponential function used to fit the PL decay curves, respectively. The fitting results of decay curves of CPCM and CPCM-TBA NCs were recorded in Supplementary Table S3. For CPCM NCs, the average PL lifetimes of exciton and Mn^2+^ emission are 2.34 ns and 1.37 ms, respectively. After the introduction of TBA, the average PL lifetimes increase to 4.81 ns and 1.58 ms, respectively. The average decay lifetime of both exciton and Mn^2+^ increases, which might be attributed to defect passivation in the NC surface by TBA addition.

**FIGURE 3:**
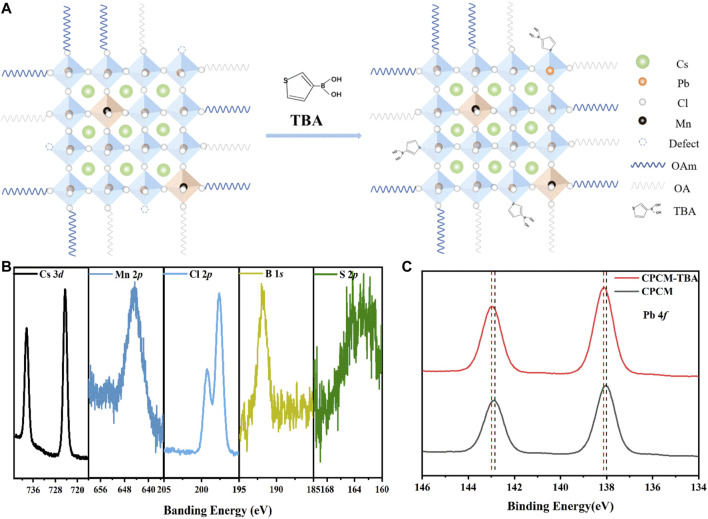
**(A)** Schematic diagram of the synthesis of CPCM NCs added with TBA. **(B,C)** High-resolution XPS spectra for Cs 3*d*, Mn 2*p*, Cl 2*p*, B 1*s*, S 2*p*, and Pb 4*f* spectra of CPCM-TBA NCs.

To further clarify the existence and function of the TBA additives, Fourier transform IR (FT-IR), ^1^H nuclear magnetic resonance (^1^H-NMR) spectra, and X-ray photoelectron spectroscopy (XPS) were applied. In the FT-IR spectra (Supplementary Figure S3), a characteristic peak of thiophene appeared at 721 cm^−1^ (Choi et al., 2021). The characteristic peaks of the thiophene are also shown in the ^1^H-NMR spectra (Supplementary Figure S4), in which δ of 7.34 and 7.12 ppm (Zhou et al., 2015; Wang Hui et al., 2020) are the resonance peaks of hydrogen on the thiophene ring. These results confirm that TBA was indeed added on the surface of CPCM NCs. In addition, we detected the peaks of S and B atoms in the XPS spectra of CPCM-TBA, which further demonstrates the existence of TBA in the CPCM NCs (Figure 3B). Meanwhile, from the XPS spectra, the Pb 4*f*_5/2_ and 4*f*_7/2_ peaks of the pristine CPCM NCs are located at 142.85 eV and 137.95 eV, respectively (Figure 3C). The peaks shift to 143.00 eV and 138.10 eV after the addition of TBA, where both peaks slightly shift approximately 0.15 eV toward the higher binding energy region. According to previous reports (Liu L. et al., 2018; Noel et al., 2020; Choi et al., 2021; He et al., 2021; Ren et al., 2021; Yuan et al., 2021), the upward shift of Pb 4*f* peaks might come from S atoms of the thiophene in TBA additives partially occupying halide vacancies and forming Pb–S bonds with Pb ions in the perovskite crystals (Noel et al., 2020). Meanwhile, it is noteworthy that the Cs 3*d* and Cl 2*p* spectra display negligible shifts upon TBA incorporation (Supplementary Figure S5). The results further indicate that TBA is not bonded with Cs^+^ or halide ions (Choi et al., 2021). In addition, the actual ratio of Mn and Pb was estimated by the relative area of XPS results (Supplementary Table S2), combining with the EDX results (Supplementary Table S1), indicating that the TBA addition shows little influence on the Mn:Pb ratio in Mn: CsPbCl_3_ NCs.

Based on these results, we propose a passivation mechanism of defects (Figure 3A). In the pristine Mn: CsPbCl_3_ perovskites, there are various structure defects (such as chloride vacancy) at the surface of the NCs (Yang et al., 2020) (Zhang Y. et al., 2021). After the addition of TBA, the under-coordinated Pb ions in the perovskite NCs form Pb–S bonds with the S atoms of the thiophene in TBA, thus passivating the surface defects and decreasing non-radiative recombination, enabling the material to improve its performance. To corroborate the versatility of TBA as ligands for perovskite NCs, we extended the NCs materials to other inorganic perovskite halides (CsPbBr_2_Cl and CsPbCl_3_), as shown in Supplementary Figure S6. The photoluminescence by intrinsic exciton radiative recombination increases with TBA addition for both CsPbBr_2_Cl and CsPbCl_3_ NCs. However, the TBA shows a more significant influence on the PL for CsPbCl_3_ than for CsPbBr_2_Cl, which may originate from CsPbCl_3_ and possess more deep-level defects such as chlorine vacancies (V_Cl_) than CsPbBr_2_Cl (Huang et al., 2016; Wei et al., 2019; Wu et al., 2019; Román-Vázquez et al., 2020). This comparison further proves that TBA ligands function as a defect passivator, especially for halide-deficient perovskite NCs.

**FIGURE 4:**
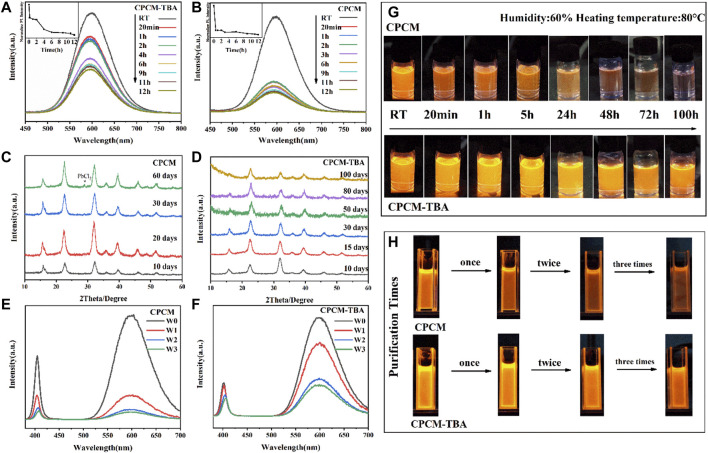
PL spectra of CPCM **(A)** and CPCM-TBA **(B)** NCs and corresponding photos **(G)** at different time intervals under heating conditions. XRD patterns of CPCM **(C)** and CPCM-TBA **(D)** NCs at different time intervals. PL spectra of CPCM **(E)** and CPCM-TBA **(F)** NCs and corresponding photos **(H)** after zero (W0), one (W1), two (W2), and three (W3) purification times. All photos were recorded under 365 nm excitation.

The stability of perovskite nanocrystals is a fundamental issue to be resolved. In order to investigate the effect of TBA ligand modification on the stability of CPCM, a systematic stability test is studied. First is the thermal stability. Figures 4A,B show the fluorescence intensity of CPCM-TBA and CPCM NC dispersed solution at 80°C under 60% humidity. During the heating process, the CPCM-TBA NCs retain approximately 50.2% of the original emission intensity after heating the samples at 80°C for 2 h. As a reference, the PL intensity of CPCM dropped to only 15% under the same condition. From Figure 4G, CPCM NCs were quenched after heating for 48 h, but CPCM-TBA still kept an obvious orange luminescence after 120 h. The thermal stability of NC films was also explored, which also has an enhancement after TBA introduction, as shown in Supplementary Figure S7. The rapid drop in PL intensities of Mn^2+^ emissions in the NCs and NC films at 80°C, probably related to the increase of non-radiative recombination centers, which due to the loss of the ligands resulted in the formation of defects at the surface of the NCs (Palazon et al., 2016; Yuan et al., 2017). The enhanced stability with TBA-modified samples might attribute to the TBA having a stronger interaction force with the surface of NCs, which makes it more difficult to be detached from the surface of NCs under heating conditions, effectively avoiding large-scale defect states on the surface of NCs. The improved thermal endurance revalidates the aforementioned positive influence of TBA toward CPCM NCs.

Second is the storage stability, manifested by XRD patterns, as shown in Figures 4C,D. The CPCM NCs appear a new peak at 28.9° after 60 days of storage, which can be attributed to the formation of PbCl_2_ from the decomposition of the CPCM, while the CPCM-TBA NCs did not show any new peaks after 100 days. Apart from the influence of defect modification, the increase of storage stability may also partially come from the enhancement of hydrophobicity (Chen et al., 2021), as shown in Supplementary Figure S8. The water contact angle increases from 66° (CPCM) to 87° (CPCM-TBA).

Third is the purification stability of NCs. Normally, the ligands on the surface of the NCs can be readily removed after the purification process, which may lead to the formation of defect/trap states, resulting in the degradation of their optical properties. The PL spectra (Figures 4E,F) and the pictures (Figure 4H) of CPCM and CPCM-TBA NCs were collected after multiple purification times. The luminescent intensity of the CPCM NCs is significantly reduced after each washing process due to ligand shedding, which led to an increase in defects. The PLQY dropped to 5% after being washed three times. In contrast, the PLQY of CPCM-TBA was still maintained at 35% after three times of purification. This might be attributed to the strong binding effect of the thiophene groups on the NC surface, and thus TBA is more difficult to be eliminated than OA and OAm in the purification process (Yang et al., 2019b). As demonstrated by the aforementioned results, TBA shows a great enhancement in the stability of CPCM NCs, which has potential for further exploration in optoelectronic applications.

**FIGURE 5:**
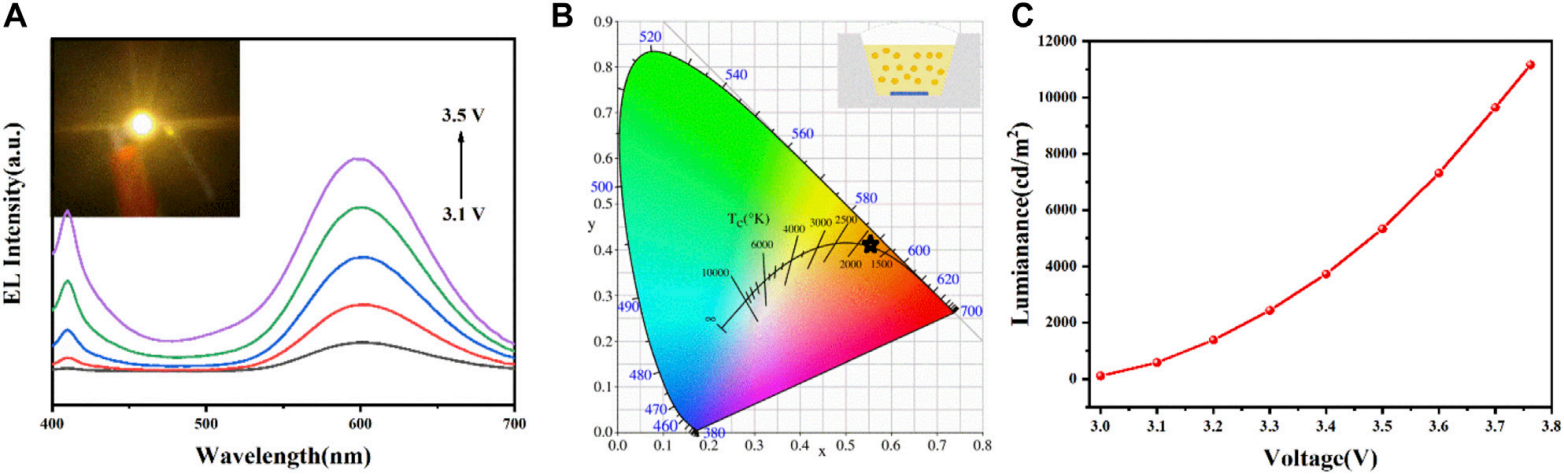
**(A)** EL spectra, **(B)** the corresponding CIE color coordinates, and **(C)** luminance–voltage curves of the LED with CPCM-TBA NCs. Inset of **(A)** shows a digital camera image of the working LED device and inset of **(B)** shows the schematic structure of the LED.

Inspired by their outstanding opto-physical characteristics and excellent stability, the orange LED devices were fabricated on commercial 365 nm InGaN UV chips. Figure 5A presents the EL spectra of the LED, which were recorded under different voltages from 3.1 to 3.5 V. The EL spectra comprised two emission peaks centered at 410 and 600 nm, where the intensities of both peaks increase gradually with the voltage. The device working on a driving voltage of 3.1 V shows bright orange emission (inset of Figure 5A). The CIE chromaticity diagram of the LED is shown in Figure 5B, and the inset presents the schematic diagram of the LED device structure. The device exhibited a CIE chromaticity of (0.5566, 0.4178) at 3.1 V, whose correlated color temperature is 2873 K. Figure 5C exhibits the luminance–voltage curve, in which a continuous luminance enhancement with the voltage increase proves high conversion efficiency of the CPCM-TBA NCs for ultraviolet light. The luminance maximum value reached 11160 cd m^−2^ at a voltage of 3.7 V. The results show that the CPCM-TBA exhibited a good performance as color conversion materials for bright orange LED devices with outstanding stability and good luminescent properties, indicating that the CPCM-TBA NCs have huge potential in LED fields.

## 4 Conclusion

In summary, for the first time, thiophene derivatives (TBA) are used as ligands to achieve high-quality Mn: CsPbCl_3_ NCs. TBA passivates the surface defects and decreases non-radiative trap states, which results in strong photoluminescence with a high PLQY of 93%. TBA shows negligible influence on the stoichiometry of the host NCs, thus affecting only the emitting strength but not the emitting position. Moreover, the TBA-decorated NCs exhibit superior thermal, shelf, and purification stability. Meanwhile, Mn: CsPbCl_3_-TBA-based LEDs with orange emission were fabricated, which show good luminescent properties of 11160 cd m^−2^ at 3.7 V. Therefore, introducing thiophene derivatives as the ligand into Mn: CsPbCl_3_ nanocrystals is a promising strategy for the application in next-generation lighting and displays.

## Data Availability

The raw data supporting the conclusions of this article will be made available by the authors, without undue reservation.
