# Supplementary material for: Thiophene Derivatives as Ligands for Highly Luminescent and Stable Manganese-Doped CsPbCl3 Nanocrystals
Source: Front Chem. 2022 Mar 1;10:849801. doi: 10.3389/fchem.2022.849801 (PMC8921502; doi:10.3389/fchem.2022.849801)
Supplement: Supplementary file 1 [file DataSheet1.docx]

Supplementary Material

Thiophene derivatives as ligands for highly luminescent and stable manganese-doped CsPbCl_3_ nanocrystals

Qian Wang^1^, Long Gao^1^, Chenxi Yu^1^, Meng Wang^1^, Lijie Gou^1^, Jiaqi Zhang^1*^

^1^ Key Laboratory of Automobile Materials, Ministry of Education, College of Materials Science and Engineering, Jilin University, Changchun 130012, China.

*** Correspondence:**Corresponding Author
[zhangjiaqi@jlu.edu.cn](mailto:zhangjiaqi@jlu.edu.cn)


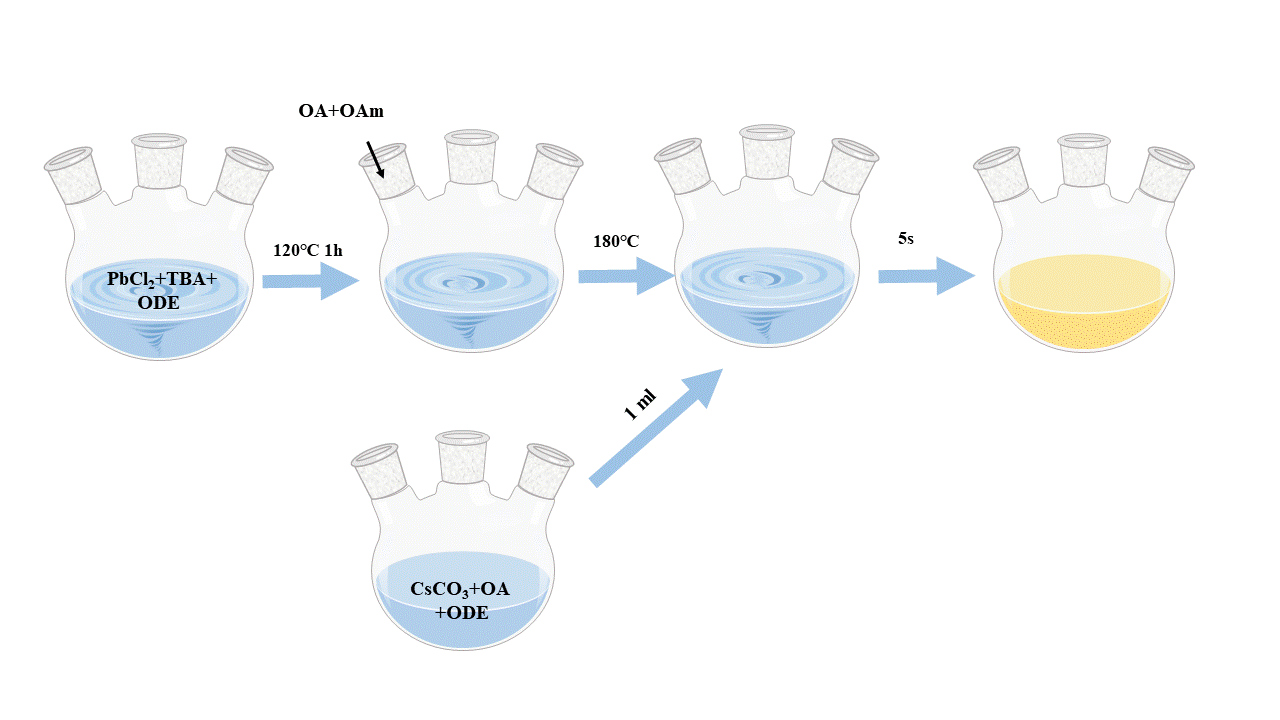


**Supplementary Figure S1** Preparation pathway of Mn: CsPbCl_3_ with TBA.


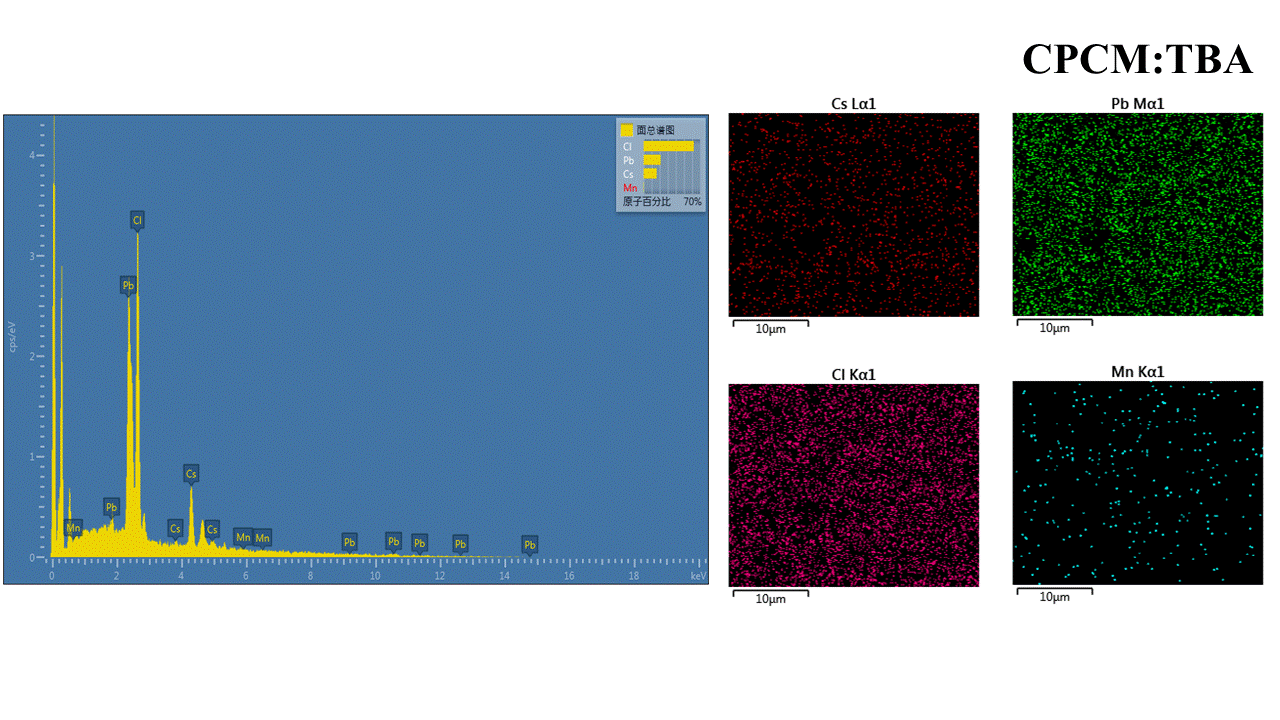

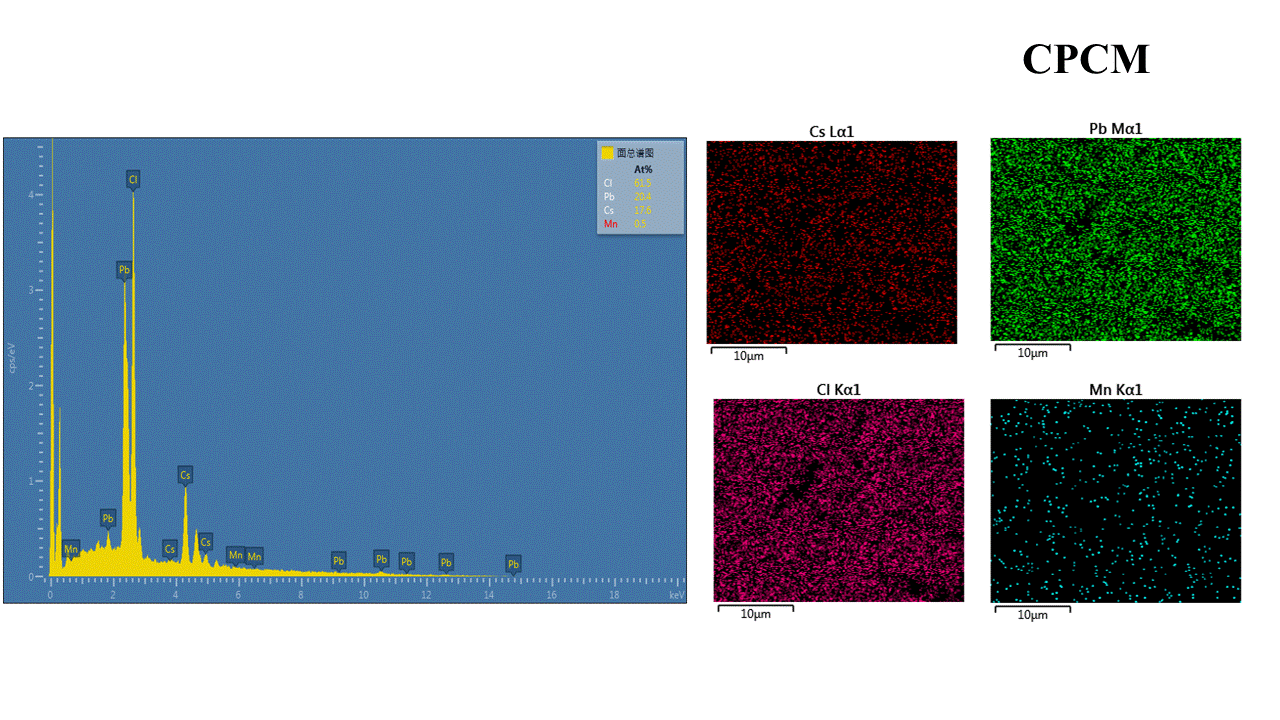


**Supplementary Figure S2** EDX measurement of the CPCM and CPCM-TBA NCs.





**Supplementary Figure S3** FT-IR spectra of Mn: CsPbCl_3_ and Mn: CsPbCl_3_ with TBA.





**Supplementary Figure S4** ^1^H-NMR spectra of Mn: CsPbCl_3_ and Mn: CsPbCl_3_ with TBA.



**Supplementary Figure S5** High-resolution XPS spectra for: Cs 3*d*, Mn 2*p*, Cl 2p XPS spectra of CPCM and CPCM-TBA NCs.

**

Supplementary Figure S6** PL spectra of CsPbBr_2_Cl with/without TBA and CsPbCl_3_ with/without TBA.


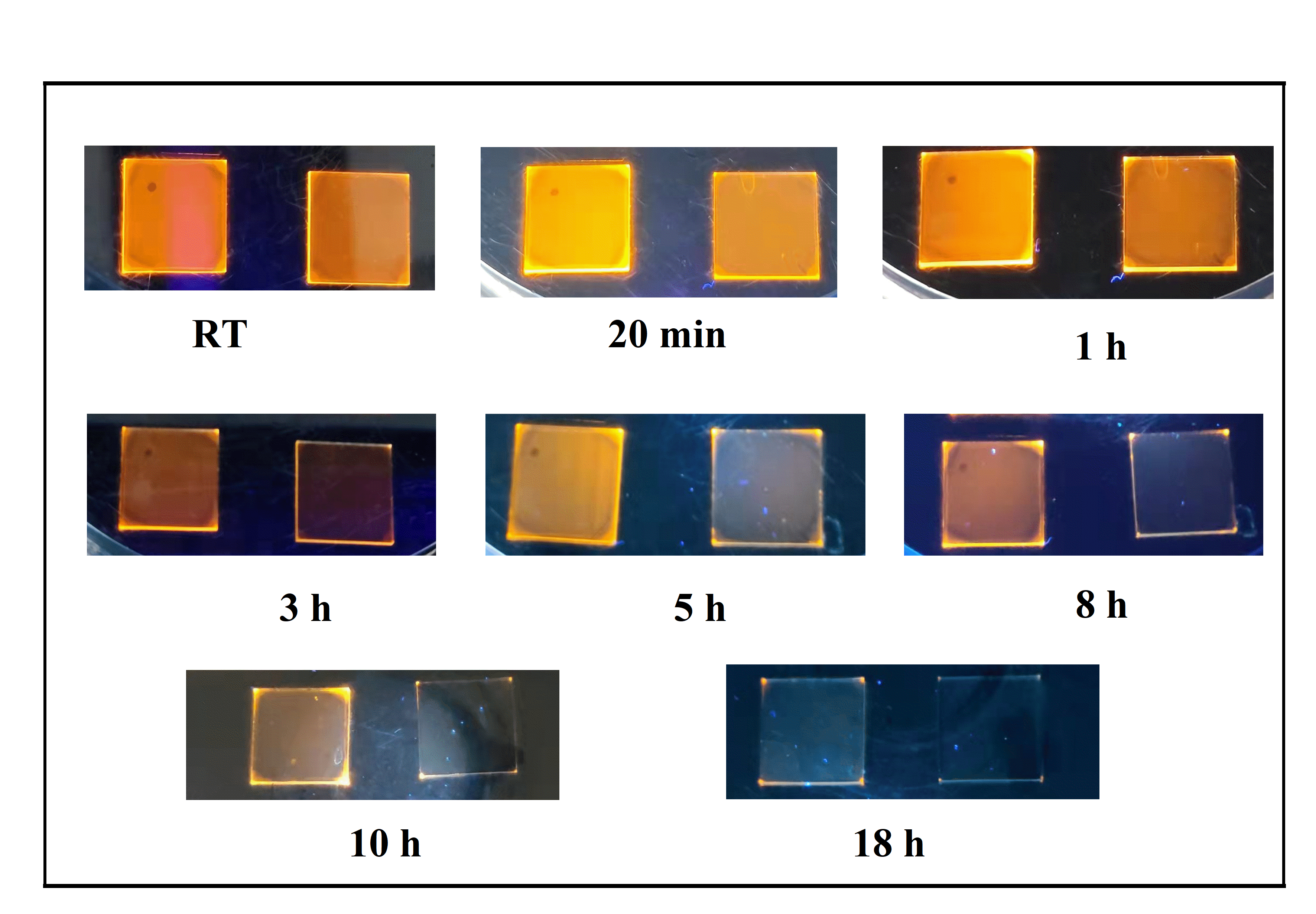


**Supplementary Figure S7** The photos of the pre-prepared plates at a successive heating process under 365 nm UV light.


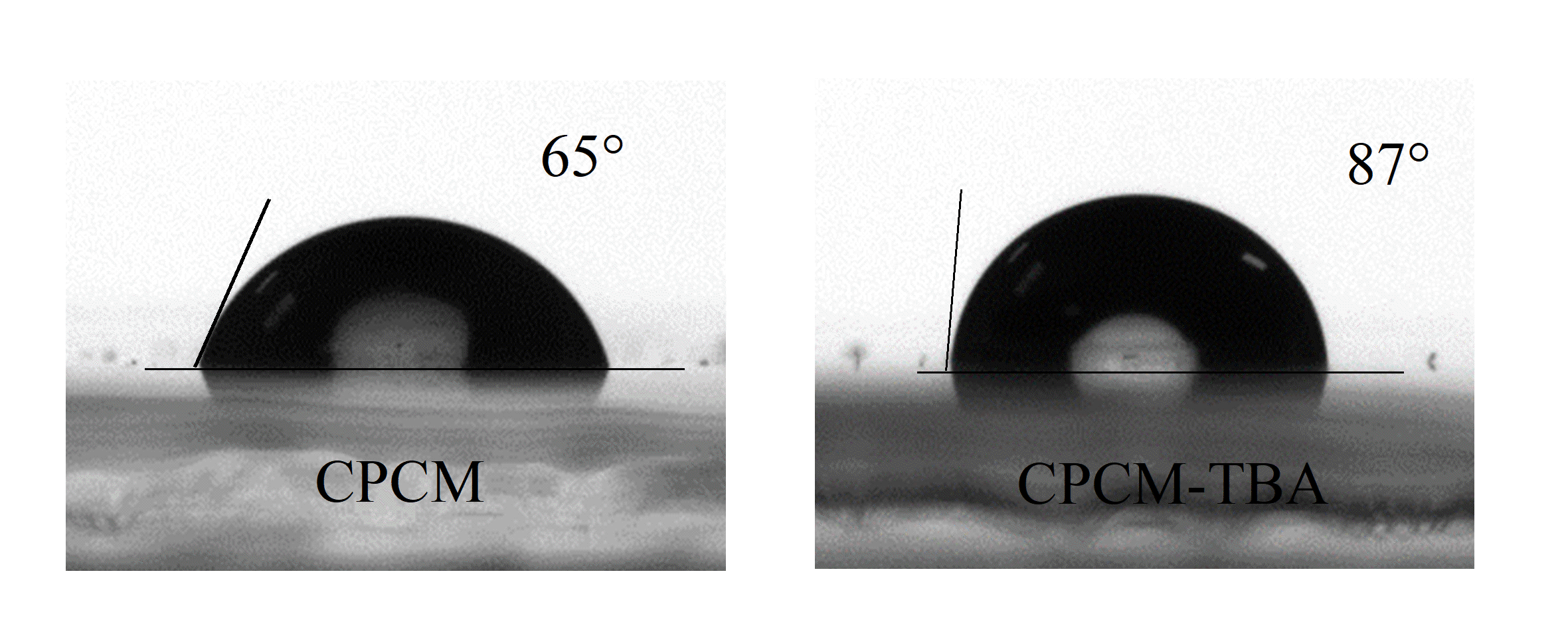


**Supplementary Figure S8** water contact angle for films before (a) and after (b) TBA addition.

**Table S1** Energy dispersive X-ray spectroscopy (EDX) results of Mn: CsPbCl_3_ and Mn: CsPbCl_3_-TBA NCs

|  | **Cs** | **Pb** | **Cl** | **Mn** |
| --- | --- | --- | --- | --- |
| Mn:CsPbCl_3_ | 17.62 | 20.39 | 61.49 | 0.50 |
| Mn:CsPbCl_3_-TBA | 16.23 | 21.15 | 62.10 | 0.52 |

**Table S2** Mn and Pb actual ratios estimated from the relative area of XPS results.

|  | Pb CPS .eV | Pb Atomic% | Mn CPS .eV | Mn Atomic% | Mn/Pb |
| --- | --- | --- | --- | --- | --- |
| Mn:CsPbCl_3_ | 69,504.25 | 86.68% | 11,314.19 | 13.32% | 0.153 |
| Mn:CsPbCl_3_-TBA | 109,454.75 | 86.05% | 18,640.48 | 13.95% | 0.162 |

**Table S****3** Fitting results of PL decay curves of CPCM and CPCM-TBA NCs.

|  |  | τ_1_ | Percent% | τ_2_ | Percent% | τ_ave_ |
| --- | --- | --- | --- | --- | --- | --- |
| CPCM | 408 nm | 0.81 ns | 75.76 | 7.14 ns | 24.24 | 2.34 ns |
| CPCM-TBA | 408 nm | 0.93 ns | 56.64 | 9.06 ns | 43.36 | 4.81 ns |
| CPCM | 600 nm | 1.37 ms |  |  |  | 1.37 ms |
| CPCM-TBA | 600 nm | 1.58 ms |  |  |  | 1.58 ms |
